# Combination Therapy of Albumin-Bound Paclitaxel and Carboplatin as First Line Therapy in a Patient with Ovarian Cancer

**DOI:** 10.1155/2014/940591

**Published:** 2014-04-03

**Authors:** K. N Srinivasan, Amit Rauthan, R. Gopal

**Affiliations:** ^1^D.M.R.T, GVN Hospital, Trichy 620008, India; ^2^GVN Institute of Oncology, 2nd Cross, Thillai Nagar, Trichy 620015, India; ^3^D.M, Manipal Hospital, Bangalore 560017, India; ^4^S.L Raheja Hospital, Mumbai 400016, India

## Abstract

*Background*. Ovarian cancer is the ninth most common cancer among women and causes more deaths than any other type of female reproductive cancer. Albumin-bound paclitaxel is known to increase intratumoral concentration of the paclitaxel by a receptor-mediated transport process across the endothelial cell wall, thereby breaching the blood/tumor interface. We present below three cases in which nab-paclitaxel based chemotherapy has been used in different settings for patients with ovarian cancer. *Case Presentation*. In the first case nab-paclitaxel was used along with carboplatin in adjuvant setting, in the second case, nab-paclitaxel was used along with carboplatin and bevacizumab as second line chemotherapy in a relapsed ovarian cancer case, and the third case delineates the use of nab-paclitaxel along with cisplatin as third line chemotherapy. *Conclusion*. In all the three scenarios, patients tolerated the chemotherapy well, as well as responding well to nab-paclitaxel based chemotherapy. The patients are currently on long-term follow-up and have been having an uneventful postchemotherapy.

## 1. Introduction

Ovarian cancer is the ninth most common cancer in women (excluding skin cancer). It ranks fifth among the causes of cancer related deaths in women worldwide. A woman's risk of getting invasive ovarian cancer in her lifetime is about one in 71 and the lifetime risk of dying from invasive ovarian cancer is about one in 95 [1]. However, there are large variations in the incidence of ovarian cancer in different areas across the world. In India, ovarian cancer is emerging as one of the most common malignancies affecting women according to the cancer registries [2]. It is one of the most lethal gynecologic diseases and lacks early detection through screening tests. The symptomatology is not very clear and, as a result, the disease is usually diagnosed at a later stage when growth has extended within the peritoneal cavity [3]. Carboplatin in combination with paclitaxel has been broadly accepted as first-line chemotherapy for advanced epithelial ovarian cancer [4]. Albumin-bound paclitaxel is known to increase intratumoral concentration of paclitaxel by a receptor-mediated transport process across the endothelial cell wall, thereby breaching the blood/tumor interface [5]. Teneriello et al. [6] have shown albumin-paclitaxel to be highly active as a single agent in patients with platinum-sensitive recurrent ovarian cancer. Albumin-bound paclitaxel is administered with 100 mL normal saline with no premedications as compared to the regular paclitaxel which required premedication, this being an added advantage in elderly diabetic women [7, 8]. The agent is well tolerated with a favorable toxicity profile. In light of the above findings, we discuss case histories of three patients presenting with ovarian cancer and treated with nab-paclitaxel.

## 2. Case 1

**Figure 1:**
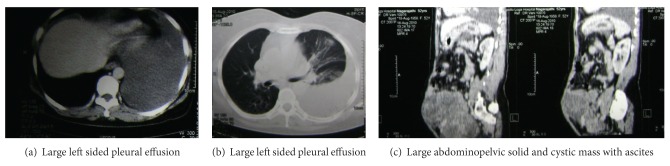
Pretreatment CT scan.

A 52-year-old postmenopausal, borderline diabetic lady presented in the second week of September 2010 with complaints like difficulty in breathing and abdominal swelling since the last 5 weeks. A chest X-ray done in July 2010 revealed left sided pleural effusion. Ultrasound of abdomen and pelvis performed in August 2010 revealed a large lesion which extended from pelvis upwards above the umbilicus which measured more than 20 cm × 14 cm × 11 cm with multiple large solid and cystic components. Following computed tomography (CT) scan reported large pelvic mass extending to left adnexal organs and left aortocaval region and a small nodule under the diaphragm segment 7 of the right lobe of liver, probably liver or peritoneal metastasis (Figure 1). Cytological examination of the ascites fluid tap done on the 20th of August 2010 revealed adenocarcinoma. The CA-125 (Cancer Antigen) level assessed on the 21st of August 2010 was 1358 U/mL (normal value is <35 U/mL).

**Figure 2:**
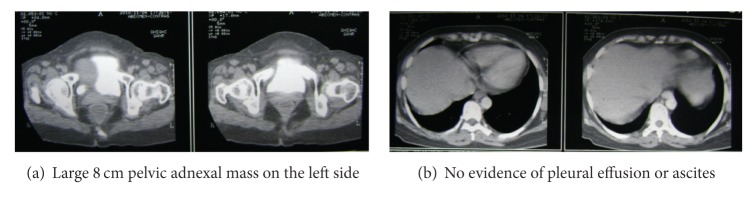
CT scan after 3 cycles of chemotherapy.

Following patient's consent, treatment was planned with albumin-bound paclitaxel 260 mg/m^2^ and carboplatin 450 mg once in 3 weeks. After the first cycle of chemotherapy, patient assessment revealed improvement in breathlessness, reduction of ascitic fluid and CA-125 levels to 392 U/mL. The chemotherapy with albumin-bound paclitaxel and carboplatin was well tolerated by the patient and there were no significant infusion related problems. Treatment was continued with two more cycles of chemotherapy. An assessment done at the end of these cycles showed a clinical improvement in the overall condition of the patient. Clinical examination revealed no ascites but a palpable mass was felt in left iliac fossa. The CA-125 level was reduced to 28.18 U/mL, and the CT scan showed a complex mass in left adnexa with normal liver and no evidence of ascites (Figure 2).

Treatment was continued with the fourth cycle of combination chemotherapy of albumin-bound paclitaxel and carboplatin. After which the patient underwent a diagnostic laparoscopy. It was observed that liver was normal. There were no intraperitoneal deposits in peritoneal cavity and omentum as well as no cystic lesions in the pouch of Douglas. Also, in both the ovaries, solid tumor elements were seen attached to sigmoid mesocolon.

Following the diagnostic laparoscopy, she underwent interval cytoreductive radical surgery and extrafascial hysterectomy along with bilateral lymphadenectomy with para-aortic sampling. Peritoneal biopsies and fluid wash were performed during the surgery. During the postoperative period no disease related events were reported. Intraoperative observations revealed a normal liver, spleen, and para-aortic regions. Bilateral ovarian tumor was adherent to uterus and sigmoid mesocolon. The histopathological finding was papillary serous cystadenocarcinoma of ovary. Pelvic lymph nodes including para-aortic regions were negative for metastasis. The peritoneal fluid wash did not indicate any features of malignancy.

**Figure 3:**
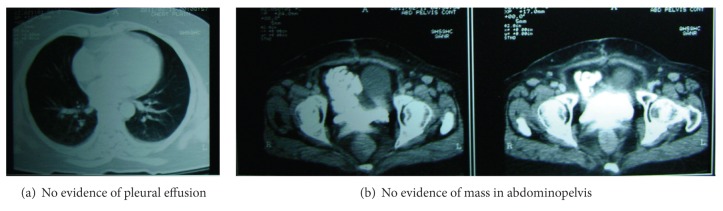
CT scan after 4 weeks following 6 cycles.

**Figure 4:**
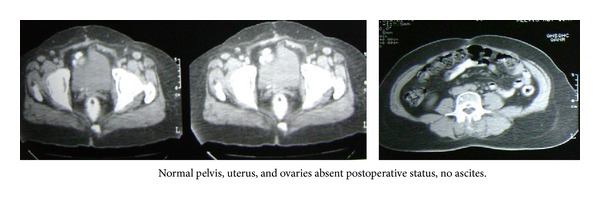
CT scan after 24 weeks following 6 cycles.

Further, the patient received two more cycles of chemotherapy with albumin-bound paclitaxel and carboplatin (fifth and sixth cycle). After completion of chemotherapy, the follow-up results showed a normal radiological finding in the chest and normal ultrasound and CT of abdomen and pelvis at both 4 (Figure 3) and 24 weeks (Figure 4) after chemotherapy. The CA-125 levels were 11.22 U/mL and 11.04 U/mL, respectively, at 4 and 24 weeks after chemotherapy.

## 3. Case 2

A 54-year-old woman was referred to our hospital in June 2008 with the history of abdominal distension, since December 2007. On examination she had a distended abdomen with umbilical hernia. Radiological investigations revealed mild ascites, ventral umbilical hernia with herniation of omentum, and thickened omentum with slight granular deposit. Analysis of the ascitic fluid revealed adenocarcinomatous cells. The CA-125 level was found to be 448 U/mL. The patient received three cycles of neoadjuvant chemotherapy with paclitaxel 175 mg/m^2^ and carboplatin AUC 6. She completed chemotherapy in April 2008. After chemotherapy, she underwent total abdominal hysterectomy with bilateral salpingo-oophorectomy and omentectomy followed by pelvic node removal in May 2008. Histology of the surgical specimen reported residual viable tumor in omentum. Both ovaries were normal. Further, three more cycles of adjuvant chemotherapy with paclitaxel and carboplatin were administered. Post adjuvant chemotherapy in August 2008, revealed that the patient was in complete response. She remained under observation and was followed up after every six months for two years.

**Figure 5:**
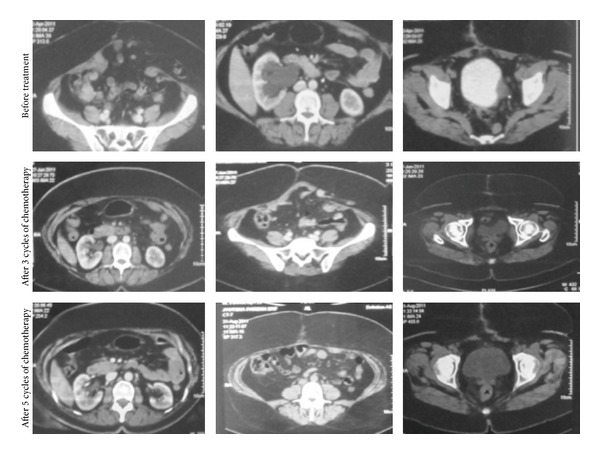
Pretreatment and after 3 and 5 cycles of chemotherapy.

In April 2011, the patient presented with complaints of burning micturition and heaviness of lower abdomen for 15 days. Her hematological parameters were within normal range but CA-125 was raised to 517 U/mL. Computerised tomography (CT) of abdomen revealed mesenteric and caecal deposit 3.5 cm × 3.5 cm. Wall infiltration was present on appendiceal surface. Another deposit in the left parametrial region, with infiltration of adjacent urinary bladder wall, enlarged right external iliac region, left gastric, and epiphrenic lymph nodes (largest lymph node measuring 18 mm). Another small deposit was observed involving the right mid-lower ureter at the iliac crossing causing moderate proximal right hydroureter and hydronephrosis. She was diagnosed with recurrent ovarian cancer. Further after obtaining consent from the patient, she was given chemotherapy with three cycles of albumin-bound paclitaxel 300 mg and carboplatin 500 mg with bevacizumab 7.5 mg/kg. After chemotherapy, the patient had neutropenia for which she was administered G-CSF 3.0 MU for 3 consecutive days. The CT done after three cycles revealed a marked reduction in the sizes of the mesenteric deposit at the caecal-appendiceal surface (17 mm) and complete resolution of left parametrial region deposit. There was a marked reduction in the size of right external iliac left gastric, and epiphrenic lymph nodes (largest residual lymph node measuring 11 mm in epiphrenic region). Also, complete resolution of small deposit involving the right mid-lower ureter at the iliac crossing with no right hydroureter and hydronephrosis, reduction in the mesenteric deposits and lymphadenopathy, and sign of good response to treatment with minimal residual disease was observed. Based on the residual disease, two more cycles of albumin-bound paclitaxel 300 mg and carboplatin 500 mg with bevacizumab 7.5 mg/kg were administered. The patient did not report and disease related events after these two cycles. The CT scan of abdomen done after completion of chemotherapy revealed complete resolution of deposits at the caecal-appendiceal surface. Right external iliac, left gastric, and epiphrenic lymphadenopathy was resolved. Complete resolution of left parametrial region deposit and deposit involving the right mid-lower ureter at the iliac crossing with resolution of the hydroureter was seen. Ca-125 levels were reduced to 4 U/mL (Figure 5).

## 4. Case 3

A 44-year-old, nondiabetic, nonhypertensive woman presented with a history of ovarian cancer, stage IIIC. She underwent primary cytoreductive surgery in November 2008. Following which, she was treated with six cycles of chemotherapy comprising of carboplatin plus paclitaxel. The patient completed chemotherapy in April 2009. After chemotherapy, her CA-125 levels were normal. After eight months, in January 2010, she complained of increasing cough and breathlessness which lasted two weeks. On investigation, her chest X-ray and CT scans revealed right pleural effusion, mediastinal lymphadenopathy, and abdominal lymphadenopathy. The CA-125 levels were increased to 600 U/mL. She was further treated with second-line chemotherapy using liposomal doxorubicin (40 mg/m^2^) and carboplatin (AUC 5) in January 2010. She had experienced toxicities such as neutropenia, fatigue, and thrombocytopenia. Subsequently, the dose of carboplatin was reduced in the fifth and sixth cycles, accordingly. She completed six cycles in May 2010. Following which, her pleural effusion was resolved and CA-125 levels reduced to 30 U/mL.

After five months, in October 2010, the patient again presented with cough and breathlessness on exertion. The chest X-ray revealed right pleural effusion and CA-125 was 216 U/mL. CT guided pleural tapping was done and she was again diagnosed with relapsed disease.

She was treated with albumin-bound paclitaxel 100 mg/m^2^ and cisplatin 60 mg weekly for 3 weeks. As the patient experienced thrombocytopenia, carboplatin was replaced with cisplatin. After chemotherapy, CA-125 levels came down to 81 U/mL. Due to thrombocytopenia, the albumin-bound paclitaxel dose was reduced to 90 mg/m^2^ in further cycles. She completed six cycles of chemotherapy in April 2011. After 3 months of follow-up, in August 2011, the CA-125 levels on assessment were found to be 40 U/mL with no evidence of pleural effusion. The patient's general condition was good and she is on a regular follow-up without recurrence since one year.

## 5. Discussion

It has been reported that the novel nanoparticle albumin-bound formulation of paclitaxel has been developed to capitalize on the transendothelial cell transport of albumin which is mediated by the glycoprotein (gp60) receptor. Albumin binds to gp60 and activates caveolin-1 which induces caveoli formation and results in invagination and pinching off of the endothelial cell membrane, thereby trapping albumin and plasma constituents in vesicular structures called caveolae. Mechanistically, this effect has been associated with higher intratumoral concentrations of paclitaxel when delivered as albumin-bound paclitaxel relative to paclitaxel [9].

Albumin-bound paclitaxel has been previously studied in platinum-sensitive recurrent ovarian cancer patients both as a single agent and in combination with carboplatin [1, 6].

The case series presented above discusses the three scenarios. In the first case, the patient received chemotherapy with albumin-bound paclitaxel and carboplatin for six cycles. After completion of chemotherapy, the follow-up results showed a normal radiological finding in the chest and normal ultrasound and CT of abdomen and pelvis. In the second case, after being treated with ovarian cancer, the patient presented with recurrent ovarian cancer. She was given chemotherapy with three cycles of albumin-bound paclitaxel 300 mg and carboplatin 500 mg with bevacizumab 7.5 mg/kg. Based on the residual disease, she received albumin-bound paclitaxel 300 mg and carboplatin 500 mg with bevacizumab 7.5 mg/kg. The patient did not report any disease related events after these two cycles. In the third case, after being treated, the patient again was diagnosed with relapsed disease. She was treated with albumin-bound paclitaxel 100 mg/m^2^ and cisplatin 60 mg. After 3 months of follow-up, in August 2011, the CA-125 levels on assessment were found to be 40 U/mL with no evidence of pleural effusion. The patient's general condition was good and she is on a regular follow-up without recurrence since one year.

A study by Coleman et al., [10] reported that nab-paclitaxel had significant activity and tolerability in a cohort of refractory ovarian cancer patients. Further, a study by Sparano et al., reported that nab-paclitaxel in combination with bevacizumab was highly effective in reducing progression free survival and overall survival [11]. These studies strengthen the evidence of usage of nab-paclitaxel of ovarian carcinoma. Hence, it can be suggested that nab-paclitaxel is used in both progressive and relapsed ovarian cancer.

## 6. Conclusion

In all the above described cases, nab-paclitaxel based chemotherapy was well tolerated by the patients. Also, the patients responded well to the therapy.
